# Evidence of synergism among three genetic variants in a patient with LMNA-related lipodystrophy and amyotrophic lateral sclerosis leading to a remarkable nuclear phenotype

**DOI:** 10.1007/s11010-021-04103-7

**Published:** 2021-03-04

**Authors:** Kathryn Volkening, Sali M. K. Farhan, Jessica Kao, Cheryl Leystra-Lantz, Lee Cyn Ang, Adam McIntyre, Jian Wang, Robert A. Hegele, Michael J. Strong

**Affiliations:** 1 Molecular Medicine, Robarts Research Institute, Schulich School of Medicine and Dentistry, Western University, London, ON Canada; 2 Department of Clinical Neurological Sciences, Schulich School of Medicine and Dentistry, Western University, London, ON Canada; 3 Analytic and Translational Genetics Unit, Center for Genomic Medicine, Massachusetts General Hospital and Harvard Medical School, Boston, MA 02114 USA; 4 Stanley Center for Psychiatric Research, Broad Institute of MIT and Harvard, Cambridge, MA 02142 USA; 5 Department of Pathology and Laboratory Medicine, London Health Sciences Centre-University Hospital, London, ON Canada; 6 Blackburn Cardiovascular Genetics Lab, Robarts Research Institute, Schulich School of Medicine and Dentistry, Western University, London, ON Canada; 7 Department of Biochemistry, Western University, London, ON Canada

**Keywords:** Amyotrophic lateral sclerosis, Fused in sarcoma (FUS), Senataxin (SETX), Lamin A (LMNA), Nuclear membrane, R-loop

## Abstract

Neurodegenerative diseases, including amyotrophic lateral sclerosis (ALS), can be clinically heterogeneous which may be explained by the co-inheritance of multiple genetic variants that modify the clinical course. In this study we examine variants in three genes in a family with one individual presenting with ALS and lipodystrophy. Sequencing revealed a p.Gly602Ser variant in *LMNA*, and two additional variants, one each in *SETX* (g.intron10-13delCTT) and *FUS* (p.Gly167_Gly168del). These latter genes have been linked to ALS. All family members were genotyped and each variant, and each combination of variants detected, were functionally evaluated in vitro regarding effects on cell survival, expression patterns and cellular phenotype. Muscle biopsy retrieved from the individual with ALS showed leakage of chromatin from the nucleus, a phenotype that was recapitulated in vitro with expression of all three variants simultaneously. Individually expressed variants gave cellular phenotypes there were unremarkable. Interestingly the *FUS* variant appears to be protective against the effects of the *SETX* and the *LMNA* variants on cell viability and may indicate loss of interaction of FUS with SETX and/or R-loops. We conclude that these findings support genetic modifications as an explanation of the clinical heterogeneity observed in human disease.

**Supplementary information:**

The online version of this article (10.1007/s11010-021-04103-7) contains supplementary material, which is available to authorized users.

## Introduction

Amyotrophic lateral sclerosis (ALS) is a fatal neurodegenerative disease that targets motor neurons and commonly results in death within 2–5 years of diagnosis. Mutations in several genes have been detected and linked to ALS. However, the disease process is quite heterogeneous, and patients with the same mutation can exhibit varying severity or progression of the disease [1, 2]. This suggests that additional genetic variants in patients may modify the disease process. Genetic examination of families with ALS (familial ALS) can be a powerful tool in determining the effect of genetic risk factors contributing to the disease. Families with individuals with different combinations of mutations in several genes that are thought to contribute to the disease can shed light into the mechanisms behind the disease by identifying which genes may be causative, or act as risk factors in developing the disease. In this study, we have identified one such family, in which multiple variants are present across family members, but only one individual is affected by ALS. This family presents with two variants in ALS-linked genes (senataxin, *SETX*, and fused in sarcoma, *FUS*), and one variant in a gene (*LMNA*; lamin A) which exhibits profound allelic heterogeneity. However, only the individual with all three variants has presented to date with ALS.

RNA binding proteins, and mutations in these proteins, appear to play a central role in ALS, including Tar DNA binding protein (*TDP-43*) [3–5], *FUS* [6–9], Rho Guanine nucleotide exchange factor (*ARHGEF28*) [10–13], mutated superoxide dismutase 1 (*SOD1*) [14, 15], senataxin (*SETX*) [16–18] and others (see [19–22] for reviews). *SETX* also displays allelic heterogeneity. Several reports have shown that deletions in *SETX* can result in Ataxia with Oculomotor Apraxia type 2 (AOA2) [23–25], ALS [26] or hereditary spastic paraplegia [27]. Juvenile onset ALS is also caused by autosomal dominant mutations in *SETX* (ALS4) [17]. Senataxin is a relatively unknown protein which appears to function by using helicase activity to aid in the resolution of R-loops, DNA/RNA hybrid structures that occur during transcription in some genes, which appear to function in preventing DNA methylation [28–31].

Variation in *FUS* have been implicated in both ALS and frontotemporal lobar degeneration [6–8, 32–34], with ALS-associated variants residing mainly in the C-terminal region near or in the nuclear localization signal [35]. These mutations appear to cause mislocalization of the protein from the nucleus to the cytosol where phase transition leads to inclusion formation [6, 36–38]. Mislocalization of FUS protein is thought to impart a toxic gain of function causing toxicity, likely in conjunction with a loss of vital nuclear function [39, 40]. Interestingly, simply altering the expression levels of FUS protein can also lead to the development of motor neuron loss suggesting a very tightly regulated level of expression is required for proper cellular homeostasis [41].

The A-type lamins belong to the family of type V intermediate filaments comprised of A-type lamins and B-type lamins. Lamin A, A∆10, C1, C2 and A∆50 (progerin) are products of one gene, *LMNA*, produced by alternative splicing events [42–48]. Lamins are integral to the structure and function of the nuclear membrane, and along with several lamin-interacting proteins contribute to shape, strength, formation, and cytoskeletal anchoring of the nuclear envelope, as well as assembly of the nuclear envelope following cell division. They are also involved in the regulation of transcription, DNA replication and DNA repair (reviewed in [48–50]). *LMNA* variants have been linked to a class of diseases known as the “laminopathies” that include the accelerated aging diseases (atypical Werner syndrome, restrictive dermopathy, Hutchinson–Gilford progeria syndrome), several muscle diseases (Emery-Dreifuss muscular dystrophy, limb-girdle muscular dystrophy, dilated cardiomyopathy and heart–hand syndrome, congenital muscular dystrophy), peripheral neuropathy (Charcot–Marie-Tooth disease) and the lipodystrophies (Dunnigan-type familial partial lipodystrophy and mandibuloacral dysplasia). Many of the variants contributing to these diseases are localized to the Ig-like fold domain of lamin A, with variants associated with one disease appearing to be clustered in a region of the protein distinct from variants associated with other disease phenotypes suggesting that specific protein regions may contribute to particular phenotypes associated with the disease [51]. There is no apparent link between ALS and variants in *LMNA*, although disruption of nuclear-cytosol movement through nuclear pores has been linked to ALS (reviewed in [52]).

In this study we have identified a family affected by ALS, with several genotypes involving variants in ALS-linked genes *SETX* and *FUS*, and a variant in *LMNA*, in which the presence of all three variants is observed only in the patient with the ALS phenotype. This suggests that co-inheritance of variants is an important risk factor in developing ALS, and that these variants likely do not function in isolation but in more complex synergistic networks which may ultimately lead to disease.

## Index case

The index case is a woman of African descent who presented to the motor neuron disease clinic at London Health Sciences Centre (LHSC) with a 6-year history of muscle cramping, a 6-month history of proximal muscle weakness, and reduced hand grip of 2 months duration. Her previous history included hypertension, hypokalemia, and a supraventricular tachycardia that had required AV nodal ablation. On examination, she demonstrated marked abdominal obesity with a body mass index of 35 kg/m^2^. In keeping with partial lipodystrophy, her arms and legs were slender with minimal subcutaneous fat. She had hypertriglyceridemia (median value 3.4 mmol/L) with dysglycemia and borderline hypertension. Her neuromuscular exam demonstrated tongue fasciculations, reduced shoulder and hand intrinsic muscle bulk, with normal reflexes. Within 6 months, reflexes had become pathologically brisk with spreading and bilateral Hoffman responses. Electromyographic studies initially demonstrated chronic motor axonal changes but within 1 year, multisegmental active denervation in the absence of sensory dysfunction was evident. At that point, 7 years following the onset of muscle cramping, a definite diagnosis of amyotrophic lateral sclerosis (ALS) was made conforming to both the El Escorial [53, 54] and Awaji [55, 56] criteria. Two years later, the patient developed bulbar dysfunction with progressive dysphagia and evidence of aspiration on a modified swallowing assessment. At the same time, she developed non-restorative sleep patterns with significant oxygen desaturations necessitating the introduction of non-invasive positive pressure ventilation. The patient remains alive, but is wheelchair bound and fully dependent for daily activities. The family history includes a total of five siblings, of whom two demonstrated long standing muscle cramping together with similar body habitus, dyslipidemia and dysglycemia in both cases. The lipodystrophy and cardiac phenotypes seen in this family were highly suggestive of a possible *LMNA* mutation while ALS mutations also needed to be examined.

## Material and methods

### Genomic DNA analysis

The coding regions and intron–exon boundaries of the *LMNA* gene were amplified, purified and genomic DNA sequence was read on an ABI 3730 Automated DNA Sequencer (Applied Biosystems, Mississauga, ON) using established protocols [57]. A variant, g.156108384G>A, p.Gly602Ser in *LMNA* was detected in the proband. We surveyed multiethnic population databases such as gnomAD [58, 59] to determine the variant allele frequency. We also further screened for the variant in 330 individuals free of any lipodystrophy or neurodegeneration and of varying ethnicities including a subset of individuals of African descent, to best match the proband’s ethnicity (*n* = 64 African, *n* = 54 East Indian, *n* = 51 Chinese, and *n* = 157 Caucasian). Briefly, this was accomplished using a rapid allele-specific detection method, using shrimp alkaline phosphatase exonuclease I treatment and ddNTP extension (SNaPshot, Applied Biosystems, Mississauga, ON), with 95% power to exclude a mutation frequency ≥ 1% in the normal population (two-tailed *α* < 0.05). Six members of the family, including the proband, were sequenced for the *LMNA* variant including four sisters and the father.

Further sequencing was performed on the proband to determine if any of the known genes associated with ALS had any variants using the next generation sequencing (NGS) based neurodegenerative disease gene panel, ONDRISeq [60]. Libraries were prepared using the Nextera Rapid Custom Capture Enrichment kit as previously described [60]. All samples were sequenced on the Illumina MiSeq Personal Genome Sequencer (Illumina, San Diego, CA, USA). 80 Neurodegenerative disease specific genes were tested using ONDRISeq [60]. ALS and FTD genes tested were: *ALS2*, *ANG*, *ARHGEF28*, *ATXN2*, *CENPV*, *CHMP2B*, *DAO*, *DCTN1*, *FI**G4*, *FUS*, *GRN*, *HNRNPA1*, *HNRNPA2B1*, *MAPT*, *NEFH*, *OPTN*, *PFN1*, *PNPLA6*, *PRPH*, *SETX*, *SIGMAR1*, *SOD1*, *SQSTM1*, *TAF15*, *TARDBP*, *UBQLN2*, *UNC13A*, *VAPB*, and *VCP* (for a full list of genes included on the ONDRISeq, see [60]). Variant calling was performed using a customized workflow within CLC Bio Genomics Workbench v6.5 (CLC Bio, Aarhus, Denmark) as previously described [60]. Variant annotation was performed using ANNOVAR with additional databases such as the Exome Aggregation Consortium (ExAC, now replaced by gnomAD) [58, 59], HGMD [61, 62], ClinVar [63], ALS Knowledge Portal [64], Project MinE data browser [65] and in-house controls. To ensure that only non-ALS samples were considered, we included the frequency of the variants within the non-neurological subset of gnomAD as the full gnomAD dataset contains exomes with a diagnosis of ALS, Alzheimer’s disease (AD), schizophrenia, and other allied neurological diseases. Variants were prioritized if they were rare (minor allele frequency < 0.1% in gnomAD), exerted non-synonymous changes or were within consensus splice sites, were previously observed in ALS and/or FTD, and had in silico values consistent with ‘damaging’ effects based on prediction outcomes of PolyPhen-2 [66], SIFT [67], CADD [68], or MPC [69]. In silico tools are not applicable to insertions or deletions. Variants were confirmed by standard PCR amplification and Sanger sequencing as previously described [60]. All available family members were screened for any genetic variants identified in the index patient using standard PCR and Sanger sequencing protocols.

**Fig. 1:**
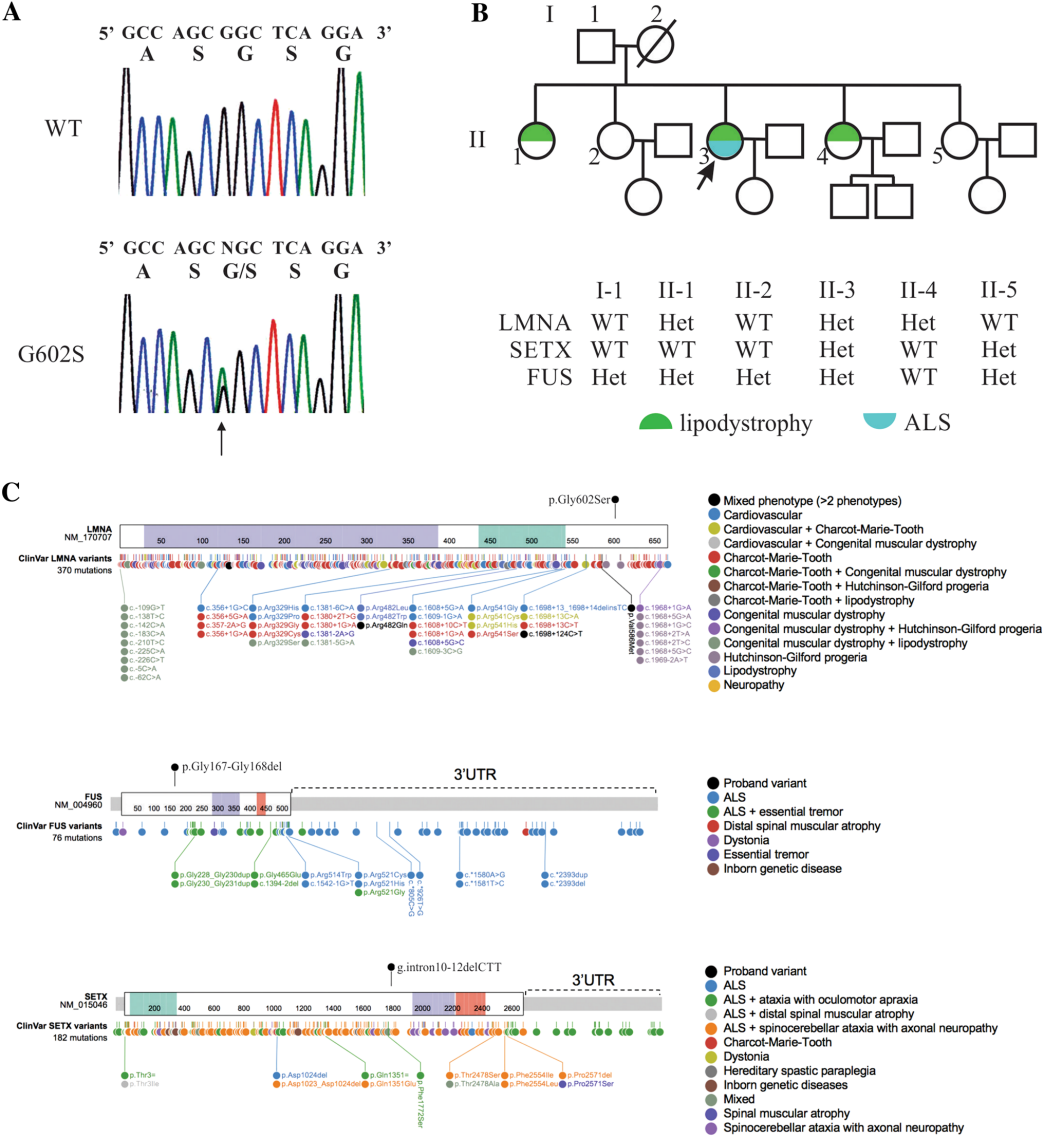
**a** Sanger sequencing of the *LMNA* gene from genomic DNA of a healthy control (WT) and the proband (G602S). The first line of text above each electropherogram tracing shows the base pair coding from sequencing, and the second lines of text show predicted amino acid sequence, **b** The family pedigree showing the proband (II-3; arrow) and genotypes and phenotypes of siblings. Each detected variant was heterozygous, and only the proband was affected by ALS. *WT* homozygotic wild type alleles, *Het* one variant and one wild type allele, males are denoted by squares, females by circles, diagonal line indicates a deceased individual, and **c** allelic heterogeneity in *LMNA*, *FUS* and *SETX* and the corresponding phenotype. Variants detected in this study are shown above each gene (black circle). LMNA purple and green domains are the intermediate filament domain and the lamin tail domain, respectively. FUS purple and red domains are the TAF15 domain and zinc finger in Ran-binding protein domain, respectively. SETX green, purple, and red domains are the SEN1 domain, AAA_1 and AAA_2 domains, respectively. ClinVar pathogenic and likely pathogenic variants are listed below each gene with clinical correlates in the legends. (Color figure online)

### Muscle biopsy

A deltoid muscle biopsy was obtained from the proband in clinic with consent under the London Health Sciences Centre (LHSC) ethics guidelines. Part of the tissue was plastic embedded and subjected to scanning electron microscopy, while the rest was fixed in 10% formalin, embedded in paraffin, and sectioned at 7 µm for immunohistochemistry. An additional muscle biopsy was obtained from a control patient under consent for comparison.

### Cloning

The pOTB-Prelamin A/C (human) vector clone was purchased from ATCC (Cat #: 7517636) and prelamin A/C amplified while incorporating *Xho*I and *Bam*HI restriction sites (For: 5′-GCC TCG AGT AGA GAC CCC GTC CCA GCG; Rev: 5′-GCG GAT CCC ATG ATG CTG CAG TTC TG). The resulting amplicon was gel purified, ligated to pGEMT-Easy (Clontech), and confirmed by sequencing. Prelamin A/C coding was then recovered *Xho*I and *Bam*HI digest, gel purified and ligated to *Xho*I/*Bam*HI digested pEGFP-C1 (Clontech). Site directed mutagenesis was then performed on pEGFP-Prelamin A/C to introduce a G>A base change at nucleotide position 1806 using site specific primers (For: 5′-GCA TCT GCC AGC **A**GC TCA GGA GCC C-3′; Rev: 5′-GGG CTC CTG AGC **T**GC TGG CAG ATG C-3′) and the QuickChange Lightning Site Directed Mutagenesis Kit (Agilent) following the manufacturer’s recommendations, creating pEGFP-G602S-Lamin (MT LMNA).

pcDNA-wild type human SETX-Flag (WT SETX) was generously provided by Dr. C. Bennett (University of California, San Diego, California, USA). pcDNA-SETX g.intron10-12,delCTT-myc (MT SETX) was purchased from GenScript (Piscataway, New Jersey, USA) using their custom construct creation service.

Wild type human FUS was amplified from RNA isolated from human spinal cord lysates (For: 5′-ATA A CT CGA GCT ATG GCC TCA AAC GAT; Rev: 5′-GTT GGT ACC TTA ATA CGG CCT CTC CCT) incorporating *Xho*I and *Kpn*I restriction sites. FUS amplicons were TA cloned to pGEM-T-Easy, and then released by *Xho*I/*Kpn*I digest (ThermoFisher) and ligated to *Xho*I/*Kpn*I double digested pmCherry-C1 (WT FUS) for confocal imaging and pEGFP-C1 for immunoprecipitation experiments. FUS p.167_168delGlyGly was cloned by deletion of nucleic acids 496–501 using the Quickchange Lightning site directed mutagenesis kit and primers containing the deletions (For: 5′-AAC AGC AGC AGT GGT GGT GGA GGT GGA G; Rev: 5′-CTC CAC CTC CAC CAC CAC TGC TGC TGT T) using either pmCherry-WT FUS or pEGFP-WT-FUS as template to yield pmCherry-MT FUS (MT FUS; for confocal experiments) and pEGFP-MT FUS (for immunoprecipitation experiments). Empty vectors pEGFP-C1, mCherry-C1 (Clontech), and pcDNA3.1-myc-his (Invitrogen) served as controls for transfections. All constructs were confirmed by sequencing.

### Tissue culture and transfections and etoposide treatment

HEK293T cells (ATCC #CRL-11268) were grown in high glucose DMEM supplemented with 10% fetal bovine serum (FBS) and penicillin/streptomycin (all from Invitrogen, Montreal, Canada) in 5% CO_2_ in air at 37 °C. Cells were transfected at 80% confluency using Lipofectamine 2000 (Invitrogen) and 2 µg of DNA in 6 well dishes according to the manufacturer’s protocol and allowed to express for 72 h before imaging. Double and triple transfections were performed in 6 well plates with 3 μg of DNA total (0.75 μg each *LMNA* and *FUS* constructs and 1.5 μg *SETX* construct) and Lipofectamine 2000 (Invitrogen) for 5 h in OptiMem (Invitrogen), followed by PBS wash, and addition of regular culture medium.

For the induction of double strand breaks (DSB) in DNA to induce R-loop formation etoposide (Millipore-Sigma cat#341205) was used [70]. A 20 mM stock solution was prepared in DMSO to make 1000 × stock. This was then diluted with culture medium to 1 × for use in culture. Cells were cultured with etoposide for 2 h to induce DSB, controls were cultured with DMSO diluted in culture medium for the same period.

### MTT assay

MTT (Sigma) was prepared as a 5 mg/ml stock in PBS. Cells were plated and transfected as above, triturated, and resuspended in 1 ml culture medium. 50 µl of this cell suspension was incubated with 10 µl of stock MTT for 30 min, cells pelleted (1000 RPM, 5 min), and the cell pellet lysed in 200 µl DMSO. This lysate was transferred to a 96 well plate and absorbance read at 570 nm. Experiments were performed in triplicate and statistical analysis performed using a non-parametric ANOVA.

### Immunocytochemistry and confocal microscopy

Cells were plated over sterilized coverslips in 6-well plates and transfected as above using mCherry-FUS, pEGFP-LMNA and myc- or FLAG-tagged SETX constructs. Constructs were expressed for 72 h in culture medium as described above. Coverslips were then washed with 1 × PBS and fixed for 15 min in 4% formaldehyde in PBS at RT, washed gently 3 times with 1 × PBS, and permeabilized with 2% Triton-X-100 in 1 × PBS for 10 min. Blocking was performed with 10% bovine serum albumin Fraction V (ThermoFisher) for 1 h at room temperature. Primary antibodies (as needed to detect SETX and endogenous proteins; see Supplemental Table 1 for all antibodies used in these studies) were then added and incubated for 1 h at RT. Cells were washed in PBS twice for 10 min each and then incubated with secondary antibody (see Supplemental Table 1) for 1 h. Cells were washed again in PBS then incubated with Hoechst for 5 min, washed twice in PBS for 5 min. For cells that did not require antibodies for detection of expressed proteins from transfected constructs, cells were fixed, washed in PBS, stained with Hoechst without permeabilization or incubation steps. Coverslips were then inverted and mounted to glass slides using Immu-Mount aqueous mounting medium (ThermoFisher) and imaged using a SP8 Leica confocal microscope.

### Immunoprecipitations and western blotting

Cells were plated to 6 well plates and transfected as described above. For some western blotting experiments, EGFP-FUS or EGFP-LMNA were co-expressed with SETX so that westerns could be performed with anti-EGFP antibodies (EGFP-FUS and EGFP-LMNA were never co-expressed in the same cells). Cells were lysed in NP40 lysis buffer [50 mM Tris pH 8.0, 100 mM sodium chloride, 1 mM EDTA pH 8.0, 1% Nonidet-P40 (Igepal), 10% glycerol, containing 1 tablet/50 ml complete protease inhibitor cocktail (Roche)]. Lysates were clarified by a 30 s spin at maximum RPM, and protein concentration determined with the DC protein assay kit (BioRad) or the Pierce protein assay kit (ThermoFisher). For whole cell lysates 50 µg of total protein was diluted in Laemmli sample buffer, denatured at 90 °C for 15 min and separated using 7.5% or 6% SDS containing polyacrylamide gels (SDS.PAGE). Proteins were transferred to nitrocellulose membrane and blocked for 1 h at RT in blocking buffer [10% skim milk powder in PBS containing 0.1% Tween-20 (PBST)]. Membranes were probed with antibodies as listed in Supplemental Table 1 in blocking buffer, then washed twice for 20 min each with PBST, then incubated with appropriate secondary antibodies (see Supplemental Table 1) for 1 h at RT in blocking buffer. Membranes were washed twice in PBST for 20 min each, then incubated with ECL reagent (Western Lightning ECL, Perkin Elmer), and visualized using a BioRad gel documentation center and ImageJ software.

For immunoprecipitations, 1 mg of total protein was subjected to immunoprecipitation (IP) using pansorbin (Calbiochem) as a carrier and 1 μg of antibody for 1 h at RT on a rotating platform. Samples were then spun at 21.1 × g, supernatant removed, and the pellet washed in lysis buffer. Washes were repeated 3 times with lysis buffer wash, and then followed by 2 washes in PBST. After the final spin pellets were fully resuspended in Laemmli PAGE sample buffer and denatured at 90 °C for 5 min. Samples were then spun to pellet the pansorbin and the supernatant run as per the western blotting protocol (below). Negative controls consisted of IP with pansorbin and rabbit IgG or mouse IgG as appropriate.

Immunoprecipitation using S9.6 antibodies methodology was modified from Cristini et al*.* [71]. Cells were not crosslinked. Lysates were sonicated for 3 × 10 s only, then subjected to IP with S9.6 antibodies with protein A Dynabeads (Invitrogen) and 10 units/ml RNAse A. As a control, RNAse H treated lysates (10 units RNAse H/ml lysate added to the IP) were used as RNAse H eliminates R-loops [72]. After washing, proteins were dissociated from the beads in 100 mM DTT by incubation at 70 °C for 10 min. Samples were then centrifuged to pellet the beads and supernatants run on either 7.5% or 6% SDS.PAGE gels and western blotted as described above.

## Results and discussion

### Co-inheritance of *LMNA*, *SETX* and *FUS* variants in a patient with lipodystrophy and ALS

Sequencing of *LMNA* from the proband was indicated due to the presence of lipodystrophy and cardiac conduction abnormalities. We detected g.156108384G>A within exon 11 in *LMNA*, producing a glycine (Gly; G) to serine (Ser; S) substitution at amino acid residue 602 (p.Gly602Ser). Sanger sequencing revealed the proband was heterozygous for the *LMNA* variant (Fig. 1a). Her surviving family members were also screened. Two sisters were found to be heterozygous for the p.Gly602Ser variant while the father and two other sisters were homozygous for wild type *LMNA* (Fig. 1b). The siblings carrying this variant were examined and presented with a similar cardiovascular phenotype as the index case with similar body habitus, dyslipidemia and dysglycemia (Table 1). The youngest (age 34) presented with a normal neurological exam. The second affected sibling demonstrated muscle cramping with reduced reflexes. The mother (putative obligate heterozygote for this variant) was reported to have the same qualitative anthropometry as the proband and her two sisters. The proband’s mother had a history of angina and there is also an extensive history of diabetes and cardiovascular disease among the relatives of the mother. None of the individuals studied were diabetic at the time of this study. Pedigree analysis is consistent with an autosomal dominant inheritance. The variant was absent from 330 normal control subjects (64 African, 54 East Indian, 51 Chinese and 157 Caucasian); however, it was observed in 70 individuals in gnomAD with an allele frequency of 0.00025. Among the 70 individuals, 60 were of African descent. When subsetting the gnomAD data to exclude individuals from the NHLBI TOPMed data, which contains many individuals with cardiovascular phenotypes, the allele frequency is maintained at 0.00021 with 57 individuals remaining, and 48 are of African descent. SIFT [67] and PolyPhen [66] each predicted this variant to be tolerated. According to ClinVar, this variant currently has a “conflicting interpretations of pathogenicity” given that several labs have labelled it as likely benign or a variant of uncertain significance. ESEfinder 3.0 [73, 74] determined that this variant did not create a new donor or acceptor site for splicing, suggesting that abnormal splicing is likely not a factor for this *LMNA* variant. Furthermore, this variant was not predicted to alter a farnesylation signal from the wild type (CGGC>CAGC) in the protein or contribute to alternate splicing through creation or abolition of a splicing donor or acceptor site. Due to these results suggesting at best a modest effect of *LMNA*, we used ONDRISeq, a neurodegeneration gene panel, to search for variation within ALS genes to explain the ALS phenotype in the proband. Two heterozygous variants were identified: a rare intronic deletion in *SETX* (g.intron10-12delCTT) and an in-frame deletion in *FUS* p.Gly167_Gly168del (Fig. 1c). The *SETX* deletion was predicted to result in mis-splicing causing non-inclusion of exon 11 (causing a deletion of aa 1792–1849) and was predicted to be deleterious (score −114.84). In contrast, the *FUS* deletion removes two glycine residues at the N-terminal region of the glycine rich domain and was predicted by Provean to be pathologically neutral. *SETX* g.intron10-12delCTT was observed in one individual in gnomAD with an allele frequency of 0.000004. Interestingly, this variant is not observed when subsetting to the non-neurological subset of gnomAD suggesting the carrier of this variant in gnomAD may have a diagnosis of ALS, Alzheimer’s disease, schizophrenia, or another allied neurological condition. Importantly, due to a lack of information on this individual’s phenotype or genomic information there is not enough evidence to speculate on the impact of this variant on the individual’s neurological disease diagnosis. This *SETX* variant was not observed in the ALS Knowledge Portal or Project MinE data browser, the two largest ALS exome and genome databases. While the *FUS* variant was not observed in gnomAD, other deletions of glycine residues within this region were observed at an elevated frequency in individuals free of neurodegeneration in keeping with a benign variant classification. Sequencing of the proband’s father revealed that he was a heterozygous carrier of only the *FUS* deletion, and while the maternal genotype could not be determined experimentally, it can be deduced that she is an obligate carrier of the *LMNA* and *SETX* variants as the proband’s siblings are also carriers.

**Table 1:** Metabolic clinical and biochemical features of family members

Individual	I-1	II-1	II-2	II-3	II-4	II-5
Age (years)	78	47	46	44	39	35
Waist (cm)		113		115	99	
BMI (kg/m^2^)		60.7		35.4	29.5	
TC (mmol/L)		5.54		4.80	3.94	
TG (mmol/L)		1.98		1.20	0.79	
LDL (mmol/L)		3.30		2.90	2.35	
HDL (mmol/L)		1.34		1.40	1.23	
Glucose (mmol/L)		5.7		5.1	5.2	
HbA1c (%)		6.4		5.6	5.9	
Insulin^a^ (mU/L)		20		11	23	
CRP (mg/L)		7.9		4.14	5.1	

Numbers correspond to the individuals in Fig. 1b. Clinical information for individuals I-1, II-2 and II-5 are not reported as they did not carry the LMNA mutation

*BMI* body mass index (< 25 kg/m^2^), *TC* total cholesterol (< 4.5 mmol/L), *TG* triglycerides (< 1.7 mmol/L), *LDL* low-density lipoprotein cholesterol (< 3.0 mmol/L), *HDL* high-density lipoprotein cholesterol (> 1.1 mmol/L), *HbA1c* glycated hemoglobin (< 5.5%), *CRP* C-reactive protein (< 2.0 g/L)

^a^Insulin (6–32 mU/L)

### Muscle biopsy analyses

**Fig. 2:**
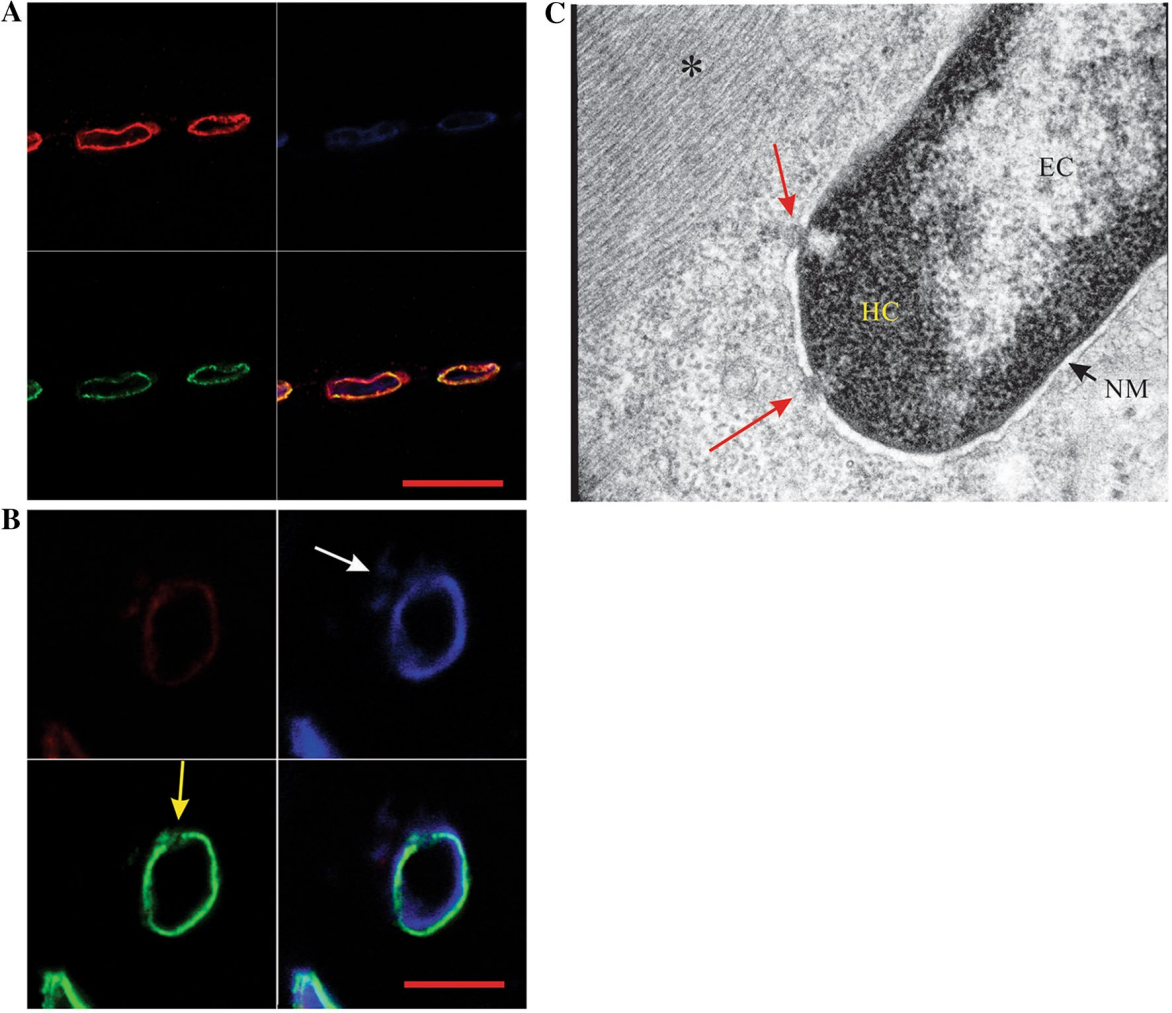
Muscle biopsies from the proband; **a** confocal micrograph of control (non-diseased) muscle tissue showing lamin (red), emerin (green) and DNA (Hoechst, blue), **b** confocal micrograph of muscle tissue from the proband showing lamin (red), emerin (green) and DNA (Hoechst, blue). The white arrow points to DNA that is located outside the nucleus in the G602S lamin muscle and a large gap in the nuclear membrane can be easily seen (yellow arrow); scale bars = 20 μm. **c** Scanning electron microscope image of skeletal muscle tissue biopsy from the proband. Regular, normal appearing myofibrils are clearly seen within the cell (*). The nuclear membrane (NM) appears to have gaps in it in two places (red arrows) in which heterochromatin (HC) appears to have been extruded into the cytosol. EC euchromatin. (Color figure online)

The deltoid muscle biopsy from the proband demonstrated both acute and chronic neurogenic atrophy with fiber type grouping. No abnormalities were noted on Gomori trichrome or PAS staining (not shown). In normal muscle tissue, both lamin and emerin immunoreactivity delineate rod-shaped nuclei in a continuous manner with no evidence of nuclear membrane interruption in normal muscle cells and chromatin is easily seen with Hoechst staining within the nuclei (Fig. 2a). In contrast, muscle from the proband (Fig. 2b) contained nuclei that were not rod shaped, but instead tended to more rounded, with clear gaps in lamin and emerin immunoreactivity (arrow, Fig. 2b). Interestingly, scanning electron microscopy of the skeletal muscle biopsy supported the observation of both gaps in the nuclear membrane and nuclear extrusion, typical of that observed in laminopathies (Fig. 2c). This material appeared to be heterochromatin and is suggestive of disruption of the integrity of nuclear membrane structure, either via failure of the nuclear membrane or in an abnormality of the nuclear pore protein complex. The combinations of mutations detected in the family were further examined in vitro to determine if there were cumulative effects causing phenotypic changes like those seen in the patient samples.

### Expression of *FUS *and *LMNA* variants in vitro is not significantly different from wild type, while *SETX* variant protein expression appears to be altered

**Fig. 3:**
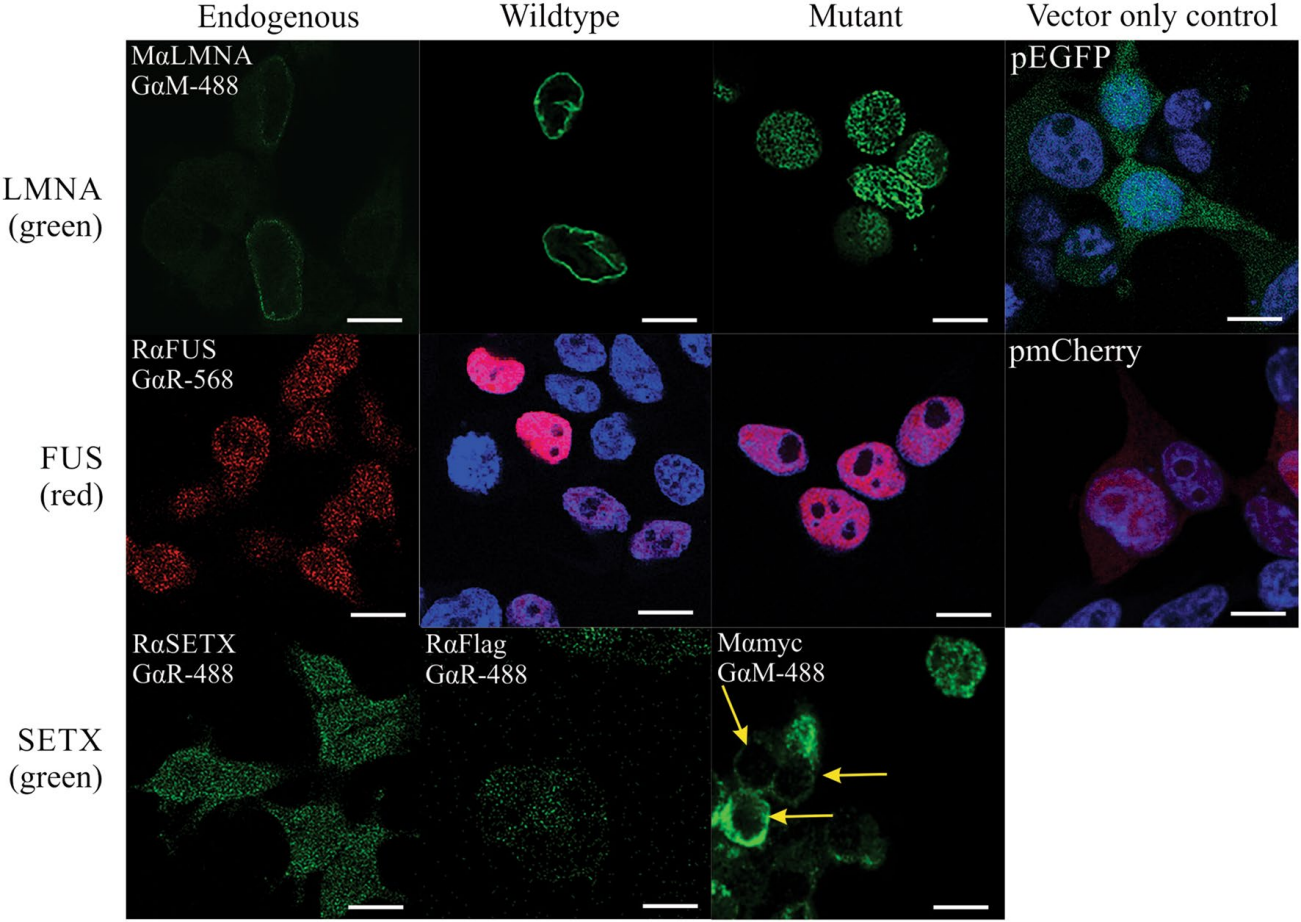
Expression of endogenous LMNA, FUS and SETX in HEK293T (left column) as detected by immunocytochemistry. Cells transfected with EGFP-WT-LMNA, pmCherry-WT-FUS and WT-SETX-Flag are shown in the second column from the left with all proteins expressed similarly to endogenous expression. Blue staining is Hoechst labelling of DNA. Cells expressing mutations are shown in the third column, while the right column shows the control EGFP and pmCherry vectors alone. Yellow arrows show altered MT-SETX-myc expression detected by immunocytochemistry using antibodies to the myc tag suggesting that the SETX mutation may lead to altered cellular localization of the protein. Scale bar = 10 μm. (Color figure online)

**Fig. 4:**
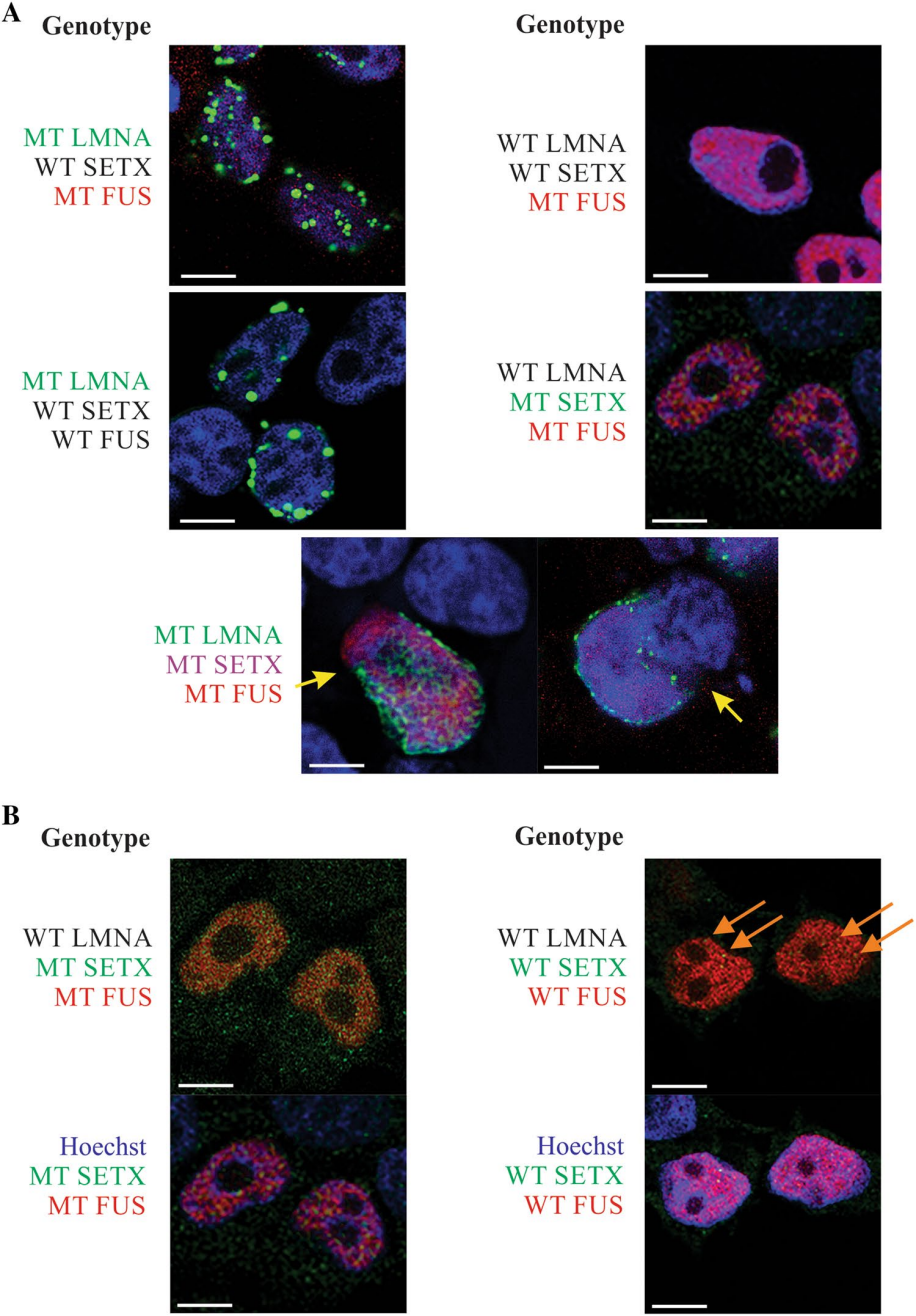
In vitro expression of variants in HEK293T cells; **a** expression of the combination of mutations detected by sequencing of the case family members. The genotype is noted beside each panel with the color denoting associated fluorescence. Genes listed in black were not detected and were the endogenously expressed wild type proteins. Co-transfection of all three mutations resulted in catastrophic breakdown of the nuclear membrane (yellow arrows) causing release of DNA (Hoechst; blue) from the nucleus and **b** MT SETX and MT FUS do not appear to colocalize while WT SETX and WT FUS appear to colocalize (yellow) in the nuclei (orange arrows). Scale bar = 10 μm. (Color figure online)

To determine the effect of the co-expression of these mutations, constructs were transfected to HEK293T cells (Figs. 3, 4). The expression of both wild type and mutated proteins was examined and compared. Additionally, MTT assays were used to determine viability of cells co-expressing the combinations of mutations as seen in the individuals in this study.

Endogenous expression of *SETX*, *LMNA* and *FUS* protein was examined in HEK293T cells to determine the normal expression of these proteins (Fig. 3). Lamin A was observed surrounding the nucleus, as expected, and as previously described [75]. Nuclear morphologies seen with *LMNA* variant expressing cells were consistent with published literature detailing that the nuclear lamina knockout or mutant cases can be folded, buckled or irregular [76, 77]. However no abnormal localization of LMNA away from one pole of the nucleus was noted in any cells, while punctate staining was observed delineating the entire nucleus (Fig. 3 top row). Endogenous FUS protein expression has been widely described and is characterized by nuclear localization with little cytosolic expression [78]. Both WT and MT FUS protein expressed from transfected constructs were also expressed mainly in the nucleus (Fig. 3 middle row). Less is known regarding the normal expression of SETX protein. In HEK293T cells, endogenous SETX protein expression detected by immunocytochemistry appears to be nuclear and cytosolic in expression (Fig. 3 bottom panel). WT SETX was detected in a similar pattern, however MT SETX appeared to have a reduction in nuclear expression in many cells, with enhanced expression in the peri-nuclear region (arrows Fig. 3, bottom panel), suggesting that the mutation may be mediating altered localization in vitro.

Co-expression of variants detected in family members was performed in HEK293T cells (Fig. 4a) to determine if there was any significant nuclear phenotype detected. As can be seen in Fig. 4a, a remarkable nuclear phenotype was detected with the combination of all three mutations (Fig. 4a, yellow arrows). Discontinuity of the nuclear membrane, as evidenced by EGFP-tagged MT LMNA was apparent, and the release of chromatin (blue; Hoechst staining) from the nucleus was also evident. This phenotype was only detected with the combination of all three mutations and was not seen when only one or two mutant proteins were expressed.

Interestingly, there was no overt co-localization of any of these proteins in the nucleus of cells with the exception of WT FUS and WT SETX (Fig. 4b). These loci were not seen in cells expressing MT FUS and MT SETX (Fig. 4b left panels).

### Cell survival (MTT) assays reveal a significant drop in viability with both wildtype and mutant protein expression, but a potential protective effect of mutant FUS when expressed with either mutant LMNA or SETX, but not both

**Fig. 5:**
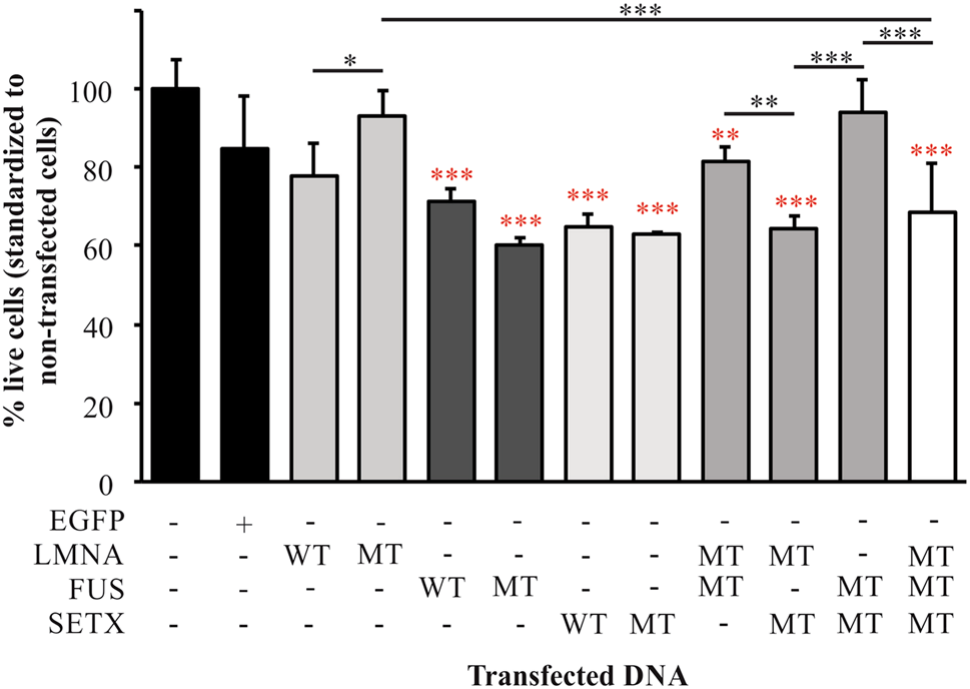
Quantification of cell viability in transfected HEK293T as measured using the MTT assay. Note the rescue of viability with the expression of MT FUS with either MT LMNA or MT SETX suggesting that the FUS mutation can alter functional outcomes of expression of either other mutation, but this protection is lost with all three mutant proteins expressed. Red * denote significance compared to EGFP transfected cells. Black * denote significance between bars as noted. *p < 0.05, **p < 0.001, ***p < 0.001. (Color figure online)

Cells were transfected with constructs for expressing WT or MT proteins alone or in combination and viability assessed using the MTT assay. Over three replicates, and using ANOVA, the significance was determined against the EGFP control transfection (Fig. 5). Untransfected and EGFP transfected cells showed no significant difference in viability. Transfection with either WT or MT LMNA did not significantly alter viability. However, consistent with other studies, expression of either WT or MT FUS had a significant deleterious effect on viability (*p* < 0.001, red * denote significance compared to EGFP in Fig. 5). Similarly, expression of WT or MT SETX also significantly decreased viability (*p* < 0.001) strongly suggesting that tightly controlled expression levels of SETX are also likely required in normal cellular homeostasis. Interestingly, the co-expression of MT FUS with MT SETX resulted in rescue of viability back to control levels, while MT FUS expressed with MT LMNA resulted in a partial rescue (*p* < 0.01) when compared to control transfections (red *, Fig. 5). MT LMNA and MT SETX expressed together had similar effects on viability as MT SETX alone, as the combination was significantly less viable than either WT or MT LMNA (*p* < 0.001) strongly suggestive that MT SETX is driving the toxicity. The co-expression of all three mutant proteins was also significantly detrimental to cell viability (*p* < 0.001) compared to control transfections, and the inclusion of MT FUS appears to have no effect on MT SETX toxicity, suggesting that MT SETX is the protein likely driving the reduced viability in this model. An alternative explanation is that the combination of MT SETX and MT LMNA expression is too detrimental for the expression of MT FUS to have any effect on viability.

### S9.6 and SETX immunoprecipitation of mutant proteins

Several of the ALS associated proteins are known to be involved in DNA/RNA binding including R-loop interaction/resolution or double stranded break repair, including FUS [79] and SETX [70, 80–82]. WT SETX is involved in the resolution of R-loops (see [83] for review). To determine if R-loop binding is altered with the mutations S9.6 antibodies were used to IP R-loop formations [71] and western blots probed for the presence of FUS, LMNA or SETX proteins.

**Fig. 6:**
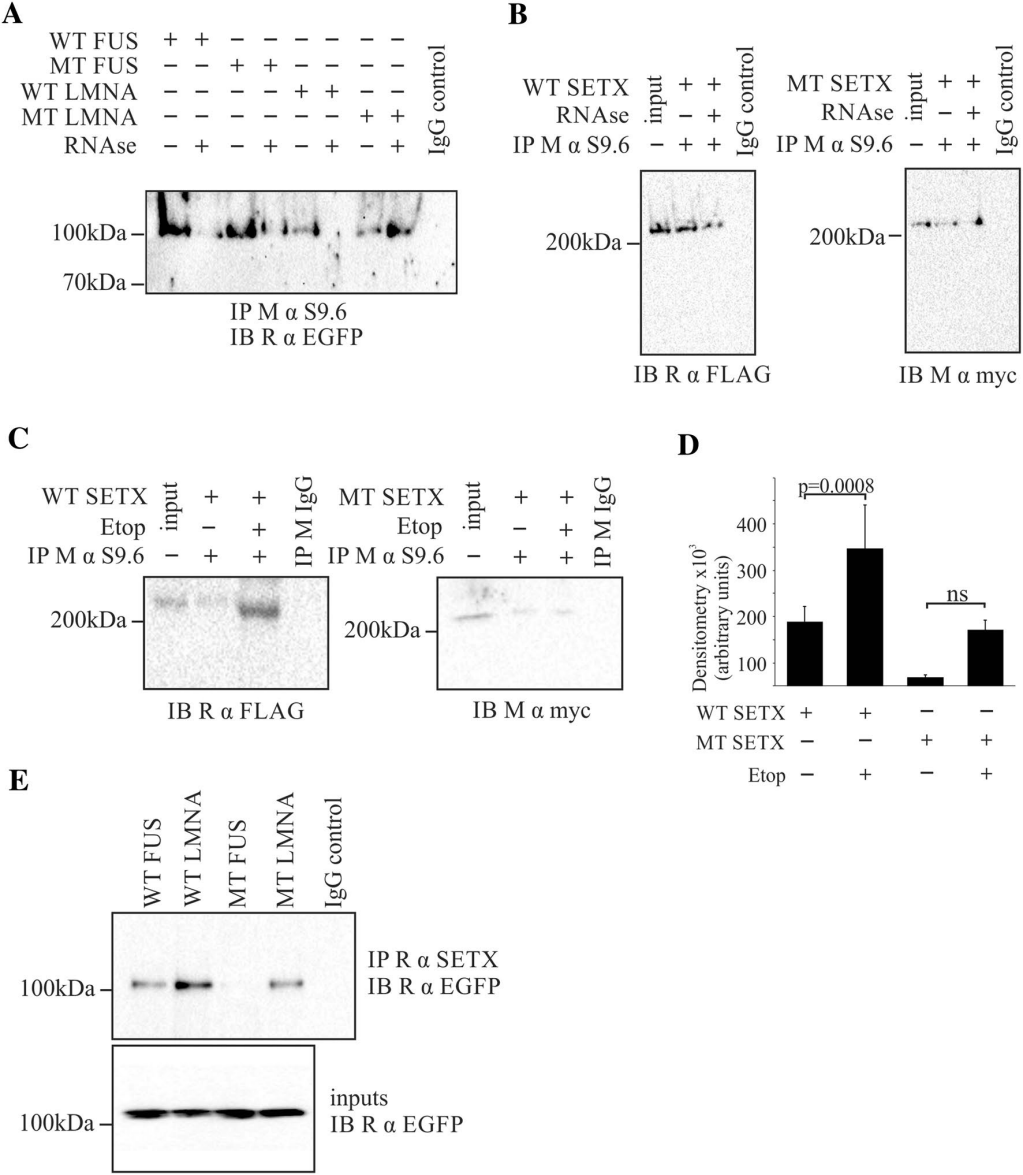
FUS and SETX interactions are altered with MT FUS. **a** WT and MT FUS are both immunoprecipitated by S9.6 antibodies, however, MT FUS is still weakly detected after R-loop degradation (RNAse H treatment) suggesting that interaction with DNA may be altered with the mutation; **b** WT SETX associates with R-loops (left panel) while MT SETX is barely detected (right panel). Input denotes 50 μg of whole cell lysate transfected with WT or MT SETX without IP as positive controls for blotting conditions (representative data from one replicate shown) and **c** etoposide treatment of cells results in increased R-loop formation, and increased WT SETX interaction with R-loops while MT SETX is barely detectable. Input denotes 30 μg of whole cells lysate transfected with WT or MT SETX without IP as positive controls for blotting conditions (representative data from one replicate shown). **d** Quantification of data from C and **e** MT FUS cannot be immunoprecipitated with WT SETX, suggesting a fundamental alteration of interaction with this mutation compared to WT FUS

WT FUS and LMNA both IP with S9.6 antibodies (Fig. 6a). Similarly, in cell lysates, both MT FUS and LMNA were detected after S9.6 IP. Lysates that were treated with RNAse H to eliminate R-loops showed minimal immunoprecipitation of WT FUS, WT LMNA and MT LMNA. Surprisingly, MT FUS was still detected (Fig. 6a) suggesting that MT FUS may interact with DNA directly, independent of the R-loop formation. WT SETX was detected with S9.6 immunoprecipitation, while signal was lost on RNAse H treatment, consistent with the localization of WT SETX with R-loops. MT SETX was extremely weakly detected with S9.6 IP (Fig. 6b, c) consistent with the localization changes seen by confocal where there appears to be decreased nuclear expression of MT SETX. Etoposide treatment was then used to induce the expression of R-loops (Fig. 6c, d) to determine if MT SETX interaction with R-loops could be more easily detected. WT SETX IP was significantly increased with treatment with etoposide (*p* < 0.0008; Student’s *t*-test compared to non-etoposide treatment), while MT SETX amounts detected were not significantly different (*p* = 0.77) upon treatment, suggesting the MT SETX is likely associated only at extremely low levels, if at all, with R-loops (Fig. 6c, d). No comparison was made between WT SETX and MT SETX due to the different tags and antibodies required to detect the proteins.

To further investigate the interaction between the three proteins, IP were performed using anti-SETX antibodies, followed by western blotting for WT or MT FUS and LMNA. Both WT and MT LMNA and WT FUS appear to co-IP with SETX, while MT FUS appears to not interact with WT SETX (Fig. 6e), suggesting that the deletion of two glycine residues in the glycine rich region has an effect on this interaction. As no colocalization between MT FUS and MT SETX was seen by confocal microscopy, the levels of MT SETX interacting with R-loops was barely detectable, and MT FUS was detected in RNAse H treated S9.6 IPs suggesting interaction with DNA directly, the interaction between MT FUS and MT SETX was not investigated.

In this study we have examined a family in which one member was diagnosed with ALS while other siblings and both parents were unaffected. While the affected individual presented with an immediate suggestion of a *LMNA* mutation based on phenotype, variants in lamin are classically not associated with ALS. They are however rarely reported in neuromuscular disorders, including in spinal muscular atrophy (p.Gln439Ter resulting in premature truncation and p.Arg337His [84, 85]), and in Charcot–Marie-Tooth disease (AR-CMT2, p.Arg298Cys [86, 87]). This warranted further genomic examinations that revealed variants in the ALS-linked genes *FUS* and *SETX*.

While the *FUS* variant (p.Gly167_Gly168del) was predicted to be non-detrimental and expression of this mutation in culture appears to be consistent with this prediction, it may have some physiological function since MT FUS appears to partially rescue MT SETX and MT LMNA phenotypes in culture and only the presence of all three variants causes the dramatic abnormal nuclear phenotype. The SETX variant (g.intron10-12, delCTT) was predicted to result in the loss of exon 11 (causing a deletion of aa 1792–1849). The function of exon 11 is unclear. However, this variant appears to affect cellular localization of SETX, its ability to associate with R-loops, and possibly its interaction with FUS.

In this family, two individuals were heterozygous for the *SETX* variant, but only one met the El Escorial criteria for diagnosis of ALS. The examination of viability confirms that the *SETX* variant is deleterious, and confocal data shows MT SETX expression is also localized to the peri-nuclear region. The reason for this is unclear and deserves further study. Of interest, the expression of MT FUS with either MT SETX or MT LMNA proteins appeared to be able to partially rescue the toxicity, and the sibling positive for *FUS* and *SETX* variants was not affected by ALS. This prompted the examination of the expression of, and interactions between, these proteins. The expression pattern of MT FUS was unremarkable, and the expression of MT LMNA paralleled other reports of lamin A variants in which cells expressing *LMNA* mutations exhibit nuclear blebbing and reduced ability to withstand shearing forces [88–93]. However, we did not observe any nuclear pole depletion of MT LMNA protein as has been described with other *LMNA* variants [76]. In the proband’s muscle biopsy, the nuclear membrane appeared to be leaking chromatin into the cytosolic space. This phenotype was recapitulated in HEK293T cells by expressing all three mutant proteins and resulted in nuclear membrane disruption with DNA release into the cytosol. This phenotype was not seen with any of the other combinations of variants found in any of the siblings. Taken together with the in vitro data, this suggests that all these variants synergistically exert their effect producing the phenotype. Whether this cooperation is through direct interaction or mediated through other interactions requires more investigation.

We hypothesize that changes in interactions seen between FUS and SETX proteins when mutations are involved is likely contributing to this phenotype, while MT LMNA is weakening the nuclear membrane to shear forces such that the combination of both these is leading to the disease state. It will be of interest to examine the interaction between FUS and SETX proteins further to determine if both of these proteins are involved in common functions concurrently, potentially in R-loop regulation as would be suggested by their colocalization and immunoprecipitation with S9.6 antibodies. SETX is known to have a role in the resolution of R loops, and while the function of R-loops is still the subject of considerable debate, they appear to be involved in the modulation of methylation of gene promoters [29, 30]. Variants in SETX may therefore alter gene methylation to some extent, depending on whether mutations increase or decrease SETX interaction with the R-loop structures [31]. Both SETX and FUS are also likely functioning in repair of double stranded DNA breaks, where R-loops tend to accumulate adjacent to the breaks [70]. FUS has also been implicated in the prevention and repair of transcriptional associated DNA damage [79] and is recruited to DSB, with ALS associated mutations interrupting this interaction [94]. The c-terminal and glycine rich domains of FUS appear to be vital to the interaction with HDAC in DNA repair [94], so mapping the minimum region required for this interaction would be of interest.

It will also be important to follow family members to determine whether a heterozygote for the *SETX* variant eventually becomes affected by ALS, since heterozygous *SETX* variants have been linked strongly to the ALS phenotype. However, to date the sibling carrying this variant remains asymptomatic. This study suggests that the combinatory effect of several variants may be important in the development of pathology in cases where single disease-predicting mutations are not present. While we cannot unequivocally determine that the combination of variants expressed in this family is the cause of ALS, and while our targeted panel approach captures most of the genes known to be involved in ALS, an exome-wide approach may reveal additional genes contributing to the development of ALS beyond the three variants reported herein. However, our in vitro data recapitulates the pathological nuclear phenotype detected in the proband, showing that expression of variants are important to the development of the nuclear pathology detected in this affected individual, and supports the current view that combinations of variants are likely of importance for clinical manifestation of disease.

## Supplementary information

Below is the link to the electronic supplementary material.

(DOCX 14 kb)

## Data Availability

Materials used in the experiments within this paper can be made available upon request, with the exception of the full-length wild type SETX construct (generously supplied by Dr. C. Bennett, University of California, San Diego, California, USA) and muscle biopsy tissue. All antibodies are commercially available.

## Abbreviations

AD: Alzheimers disease
ALS: Amyotrophic lateral sclerosis
*ALS2*: Alsin2
*ANG*: Angiogenin
AOA2: Ataxia with Oculomotor Apraxia type 2
*ARHGEF28*: Rho guanine nucleotide exchange factor 28
*ATXN2*: Ataxin2
*CENPV*: Centromere protein V
*CHMP2B*: Charged multivesicular body protein 2B
*DAO*: d-amino acid oxidase
*DCTN1*: Dynactin subunit 1
DMSO: Dimethyl sulfoxide
DSB: Double strand breaks
EC: Euchromatin
ECL: Epichemiluminescence
EDTA: Ethylenediaminetetraacetic acid
EGFP: Enhanced green fluorescent protein
*FIG4*: FIG4 phosphoinositide 5-phosphatses
*FUS*: Fused in sacrcoma
*GRN*: Granulin precursor
HC: Heterochromatin
HEK: Human embryonic kidney
Het: Heterozygous
*HNRNPA1*: Heterogeneous nuclear ribonucleoprotein A1
*HNRNPA2B1*: Heterogeneous nuclear ribonucleoprotein A2B1
IP: Immunoprecipitation
*LMNA*: Lamin A
*MAPT*: Microtubule associated protein tau
MT: Mutant
MTT: (3-(4,5-Dimethylthiazol-2-yl)-2,5-diphenyltetrazolium bromide
*NEFH*: High molecular weight neurofilament
NM: Nuclear membrane
*OPTN*: Optineurin
*PFN1*: Profilin1
*PNPLA6*: Patatin like phospholipase domain containing 6
*PRPH*: Peripherin
SDS.PAGE: Sodium doedecyl sulphate polyacrylamide gel electrophoresis
*SETX*: Senataxin
*SIGMAR1*: Sigma non-opioid intracellular receptor 1
*SOD1*: Superoxide dismutase 1
*SQSTM1*: Sequestosome 1
*TAF15*: TATA-box binding protein associated factor 15
*TARDBP*, TDP-43: TAR DNA binding protein of 43 kDa
*UBQLN2*: Ubiquilin2
*UNC13A*: Unc-13 homolog A
*VAPB*: VAMP associated protein B and C
*VCP*: Valosin containing protein
WT: Wild type

## Code availability

NA.
