# Supplementary material for: Evidence of synergism among three genetic variants in a patient with LMNA-related lipodystrophy and amyotrophic lateral sclerosis leading to a remarkable nuclear phenotype
Source: Mol Cell Biochem. 2021 Mar 4;476(7):2633–50. doi: 10.1007/s11010-021-04103-7 (PMC8192393; doi:10.1007/s11010-021-04103-7)
Supplement: Supplementary file 1 — (DOCX 14 kb) [file 11010_2021_4103_MOESM1_ESM.docx]

**Supplemental Table 1** Antibodies used in this study

| Antibody | Use (dilution) | Company | Catalogue Number |
| --- | --- | --- | --- |
| M α S9.6 | IP (1ug/ml) | Abcam | ab234957 |
| R α FUS | IP (1ug/ml), ICC-P (1:1000); WB (1:5000) | ProteinTech | 11570-1-AP |
| M α FUS | IP (1ug/ml); WB (1:5000) | Abnova | H00010772-M03 |
| M α LaminA | WB (1:2000) | Abcam | ab8980 |
| R α LaminA/C | IP (1ug/ml); ICC-P (1:500); WB (1:5000) | Abcam | ab68417 |
| R α SETX | IP (1ug/ml); ICC-P (1:1000); WB (1:2000) | Invitrogen | PA5-72986 |
| R α emerin | ICC-P (1:500) | Abcam | ab40688 |
| R α FLAG | ICC-P (1:5000) | Thermo | PA1-984B |
| M α myc | ICC-P (1:1000); WB (1:5000) | Cedarlane | CLX229AP |
| G α M-HRP | WB-S (1:5000) | BioRad | 170-6516 |
| G α R-HRP | WB-S (1:5000) | Invitrogen | 65-6120 |
| G α M AF-488 | ICC-S (1:1000) | Invitrogen | A-11029 |
| G α R AF-488 | ICC-S (1:1000) | Invitrogen | A-11008 |
| G α R AF-633 | ICC-S (1:1000) | Invitrogen | A-21070 |
| G α R AF-546 | ICC-S (1:1000) | Invitrogen | A-11035 |
|  |  |  |  |

AF=Alexafluor; G=goat; HRP=horseradish peroxidase; ICC=immunohistochemistry; IP=immunoprecipitation; M=mouse; R=rabbit; P=primary; S=secondary; WB=western blot
